# Obesogenic effects of warm temperature involve feeding adaptation by preoptic area leptin receptor neurons

**DOI:** 10.21203/rs.3.rs-7032725/v1

**Published:** 2025-08-21

**Authors:** Laura Kaiser, Nathan Lee, Jaclyn Williams, Michael Smith, Robert C. Noland, Sangho Yu, Christopher D Morrison, Hans-Rudolf Berthoud, Heike Münzberg

**Affiliations:** 1 Neurobiology of Nutrition and Metabolism Department, Pennington Biomedical Research Center (PBRC), LSU system, Baton Rouge, LA, USA

**Keywords:** melanocortin-4-receptor, fasting, meal pattern, satiety, energy-state

## Abstract

The preoptic area (POA) is a well-established regulator of body temperature, but its role in feeding behavior remains underexplored. Our study identifies leptin receptor (Lepr)-expressing neurons in the POA (POA^Lepr^) as critical component to suppress food intake (FI) and increase satiety in response to warm ambient temperatures. Utilizing chemogenetic activation in mice of both sexes, we demonstrate that selective activation of POA^Lepr^ neurons mimics the effects of warm temperatures, leading to a significant reduction in FI. POA^Lepr^ neurons project to the melanocortin pathway, where activation of melanocortin-4 receptors (MC4R) also suppresses FI in a temperature-dependent manner. Our findings suggest that POA^Lepr^ neurons integrate thermal and metabolic cues, demonstrating that ambient temperature is an integral part of body weight homeostasis by modulating meal size and satiety via POA^Lepr^ neurons. These results offer new insights into the neurochemical and functional properties of POA functions, expanding the traditional view that the POA is exclusively involved in thermoregulation and underscoring its broader role in energy balance.

## Graphical Abstract

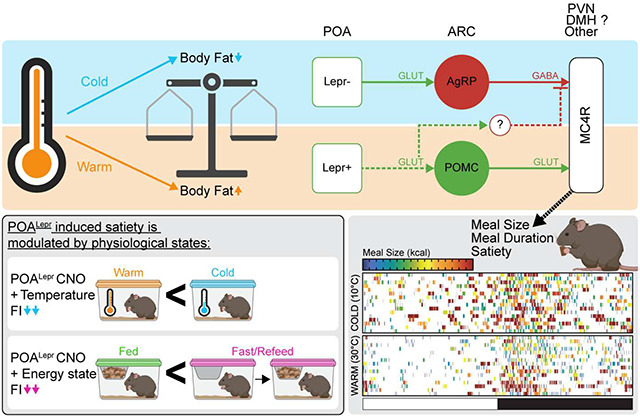

## 1. Introduction

With obesity rates continuing to rise globally, there is an urgent need to identify the neural circuits that integrate environmental and metabolic cues to develop more effective strategies for prevention and treatment. Body weight homeostasis depends on a dynamic balance between FI and energy expenditure (EE), processes governed by distinct but interacting neural pathways. Circuits that mediate changes in FI have been extensively studied in the context of energy availability – such as during fasting and refeeding – but far less is known about how the brain adjusts feeding behavior in response to other physiological challenges, including ambient temperature. Thermoregulatory circuits – particularly those driving thermogenesis and EE – have primarily been studied in the context of cold exposure or fever and are known to involve the POA of the hypothalamus^1^. Despite its central role in temperature regulation, the POA has not been traditionally recognized as a regulator of FI or body weight (BW)^2^.

The POA contains distinct cold- or warm-activated neurons that both express the vesicular-glutamate-transporter-2 (POA^Vglut2^)^3,4^. Recent work has shown that POA^Vglut2^ neurons influence temperature-dependent feeding behavior. Bulk POA^Vglut2^ activation overall suppresses FI, while projection-specific activation revealed a mix of orexigenic and anorexigenic responses^3^. Similarly, bulk activation lowers body temperature – a warm-adaptive response – yet increases nesting behavior, which is typically associated with cold exposure^3^. These findings suggest that simultaneous activation of both warm- and cold-responsive POA^Vglut2^ populations drive a complex and competing blend of thermoregulatory behaviors^5^, complicating the interpretation of how warm-specific circuits regulate FI.

Our lab previously identified POA^Lepr^ neurons as a distinct population of warm-sensing POA neurons^6^. POA^Lepr^ neurons are embedded within classic thermoregulatory circuits that not only suppress thermogenesis but also potently inhibit FI and promote BW loss^6,7^. Importantly, we and others have shown that warm-activated POA neurons, and specifically POA^Lepr^ neurons, are glutamatergic^6,8–10^, a notable shift from earlier models that considered warm sensing POA neurons primarily as GABAergic^11,12^. This highlights Lepr expression as an effective and selective marker for targeting a uniform, warm-activated neuronal population within the POA. By leveraging POA^Lepr^ neurons, we can specifically investigate how warm ambient temperatures suppress FI without the confounding influence of cold-activated circuits.

Downstream of the POA, the arcuate nucleus (ARC) of the hypothalamus is a critical hub for energy sensing and body weight regulation, integrating signals related to energy balance through melanocortin pathways involving anorexigenic Pro-opiomelanocortin (POMC) and orexigenic Agouti-Related Protein (AgRP) neurons^13^. POMC neurons are activated during refeeding to promote satiety, whereas AgRP neurons are activated during fasting to stimulate food-seeking and feeding behaviors^14,15^. Recent studies have implicated the ARC in temperature dependent FI, demonstrating that AgRP neurons are required for cold-induced hyperphagia^3,4,16^. Activation of POA^Vglut2^ projections to the ARC predominantly drive cold-induced FI responses^3,4^, likely through the recruitment of AgRP neurons. However, the ARC also contains POMC neurons that express warm-sensitive receptors^17^, and activation of MC4R seems essential for suppressing FI at warm ambient temperatures^18^. These findings suggest that activating mixed populations of warm- and cold-responsive POA^Vglut2^ projections to the ARC may mask the contribution of warm-sensing circuits, such as POMC neurons and downstream MC4R signaling.

In contrast, activation of POA^Vglut2^ projections to other melanocortin pathway nodes, including the paraventricular hypothalamus (PVN) and dorsomedial hypothalamus (DMH), results in FI suppression consistent with responses observed during warm exposure^3^. These findings underscore the complex and region-specific ways in which the POA engages the melanocortin feeding pathway. Whether the POA modulates FI through direct projections to regions that modulate FI in both directions, like the PVN and DMH, or indirectly through modulation of ARC neurons remains an important and unresolved question.

The current study defines POA^Lepr^ neurons as critical mediators of warm temperature-induced suppression of FI and identifies downstream circuits that contribute to this effect. Through selective activation of Lepr-expressing neurons within the POA, we demonstrate that POA^Lepr^ neurons are sufficient to mimic the feeding suppression observed during warm exposure. Our findings further establish that glutamatergic POA^Lepr^ neurons integrate thermal and metabolic signals to regulate BW and engage downstream melanocortin nodes, where MC4R activation further suppresses FI in a temperature-dependent manner. Together, these results position POA^Lepr^ neurons as a central link between thermoregulation and feeding pathways, expanding their role in FI control and energy balance regulation.

## 2. Results

### Temperature-dependent adaptation of energy expenditure and food intake induce changes in body weight set point.

**Figure 1:**
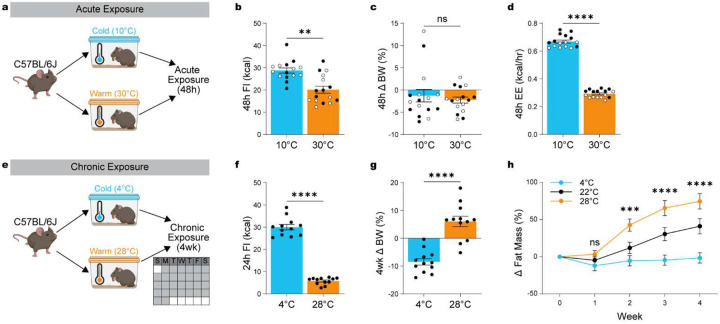
Temperature-dependent adaptation of energy expenditure and food intake alters body weight set point. (**a**) Schematic overview of acute 48h exposure to warm (30°C) versus cold (10°C) ambient temperatures in male and female mice (n = 15–16, 7–8 males, 8 females). (**b**) 48h food intake. (**c**) 48h change in body weight (%). (**d**) Average energy expenditure during 48h exposure. (**e**) Schematic overview of chronic 4wk exposure to cold (4°C) or warm (28°C) temperatures over four weeks (n = 12, all males). (**f**) 24h food intake at the end of 4wk exposure. (**g**) 4wk change in body weight (%). (**h**) Change in fat mass accumulation. Data are presented as mean ± SEM. *p < 0.05, **p < 0.01, ***p < 0.001 (t-test for A-D, two-way ANOVA with Bonferroni’s post hoc test for E). Males are represented by black circles, females by white circles.

We first confirmed the robust physiological adaptations in EE and FI elicited by acute changes in ambient temperature by housing mice at either cold (10°C) or warm (30°C) temperature for 48h (Figure 1a). As shown previously^6^, exposing mice to warm and cold temperature caused significant changes in FI (Figure 1b; paired t test: t_(15)_ = 3.33, p = 0.0049) but not in BW (Figure 1c; paired t-test: t_(15)_ = 0.69, p = 0.5034), due to simultaneous energetic balancing with suppressed EE (Figure 1d; paired t test: t_(15)_ = 47.00, p < 0.0001).

While these acute adaptations are well-documented, the long-term consequences on BW have received less attention. To address this, we assessed whether chronic housing at cold (4°C) or warm (28°C) temperatures over four weeks alters BW homeostasis (Figure 1e). As expected, sustained warm exposure led to a decrease in FI (Figure 1f; independent t test: t_(22)_ = 18.89, p < 0.0001). Notably, chronic temperature also significantly altered BW (Figure 1g, independent t-test: t_(22)_ = 6.604, p < 0.0001) and fat mass (Figure 1h). Cold exposure (4°C) prevented the normal gain in fat mass seen in mice housed at RT (22°C), whereas warm exposure (28°C) significantly increased fat mass (Figure 1h; repeated-measures ANOVA for the effect of temperature during exposure: F_(8, 124)_ = 15.75, p < 0.0001).

These findings establish that ambient temperature serves as a potent exteroceptive cue that impacts energy balance beyond immediate thermogenic responses. Despite suppression of FI, the concurrent suppression in EE seems to drive BW gain and adiposity, underscoring the importance of FI and EE balance in temperature-driven metabolic adaptations. While many studies have addressed EE in thermoregulatory control, the neural circuits responsible for warm-induced suppression of FI remain unexplored.

### Chemogenetic activation of POA^Lepr^ neurons mediates temperature-dependent adaptations of feeding behavior.

Previous studies have shown that POA^Lepr^ neurons exhibit warm-sensing properties^6,8–10^ and modulate EE in a temperature-dependent manner^6^. Moreover, chemogenetic activation of POA^Lepr^ neurons decrease FI^6^, mimicking the natural effects observed during warm exposure. Based on these findings, we hypothesized that activating POA^Lepr^ neurons would induce a temperature-dependent suppression of FI.

**Figure 2.**
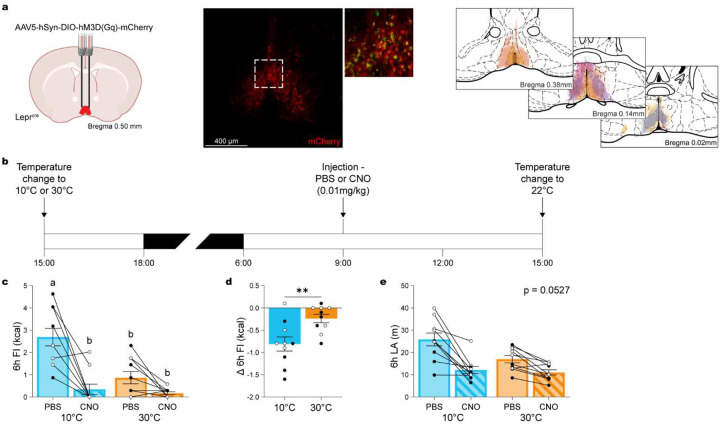
Activation of POA^Lepr^ neurons mediates temperature-dependent feeding adaptations. (**a**) Schematic showing AAV5-hSyn-DIO-hM3D(Gq)-mCherry injection into the POA of Lepr^Cre^ mice, with a representative histological image confirming viral spread and viral injection maps. Each color represents an individual animal. (**b**) Experimental timeline assessing the effects of chemogenetic activation of POA^Lepr^ neurons with CNO (0.01 mg/kg, i.p.) or PBS during 24-hour exposure to cold (10°C) or warm (30°C) temperatures (n = 10, 5 males, 5 females). (**c**) Total food intake over 6 hours. (**d**) Change in 6-hour food intake, demonstrating temperature-dependent suppression of feeding following POA^Lepr^ activation. (**e**) Locomotor activity (LA) remains unchanged across conditions. Data are presented as mean ± SEM. Different letters above bars indicate statistically significant differences between groups. *p < 0.05, **p < 0.01, ***p < 0.001 (two-way ANOVA with Bonferroni’s post hoc test for C and E, t-test for D). Males are represented by black circles, females by white circles.

To test this, we selectively expressed DREADD-Gq in POA^Lepr^ neurons of Lepr^cre^ mice (Figure 2a), enabling synthetic activation of these neurons. As shown previously^6^, POA^Lepr^ neurons are highly sensitive to chemogenetic stimulation, resulting in suppressed EE, decreased core body temperature, and reduced locomotor activity (LA) under thermoneutral conditions. We initially evaluated different doses of clozapine *N*-oxide (CNO; i.p.) and identified 0.01 mg/kg as the lowest dose that robustly reduced EE, with effects beginning to wane within 3 hours (Figure S1a; ANOVA time/treatment interaction: F_(1440, 8640)_ = 3.668, p < 0.0001). While this dose significantly reduced 6-hour LA (Figure S1b; repeated-measures ANOVA: F_(5, 30)_ = 33.23, p < 0.0001), LA and FI were not significantly correlated (Figure S1c; correlation: r_(8)_ = 0.4495, p = 0.2638). Thus, we used 0.01 mg/kg CNO for subsequent experiments to maximize effects on EE while minimizing LA-related confounds.

Mice were then exposed overnight to either 10°C or 30°C to physiologically silence or activate POA^Lepr^ neurons, respectively^6^, followed by injection with CNO (0.01 mg/kg, i.p.) or PBS the next morning (Figure 2b). At 10°C, CNO significantly suppressed FI to levels comparable to natural suppression observed at 30°C (Figure 2c; ANOVA temperature/treatment interaction: F_(1, 36)_ = 9.369, p = 0.0042). In contrast, when POA^Lepr^ neurons were already physiologically active at 30°C, CNO had no significant effect on FI. This temperature-dependent effect was further supported by comparing net CNO-induced FI changes at 10°C versus 30°C, which revealed significantly greater suppression at 10°C (Figure 2d; paired t-test: t_(9)_ = 3.749, p = 0.0046). These findings demonstrate that POA^Lepr^ neurons mediate acute, temperature-dependent suppression of FI.

To rule out the possibility that FI suppression was secondary to reduced LA, we again examined LA responses. Although there was a trend towards CNO decreasing LA at 10°C, the interaction between treatment and temperature did not reach significance (Figure 2e; ANOVA for the interaction of temperature and treatment: F_(1, 36)_ = 4.015, p = 0.0527). While this suggests a contribution of LA to the observed FI effects, the lack of significant interaction indicates that LA is unlikely to be the primary driver.

### Temperature-dependent adaptations in meal patterns.

**Figure 3:**
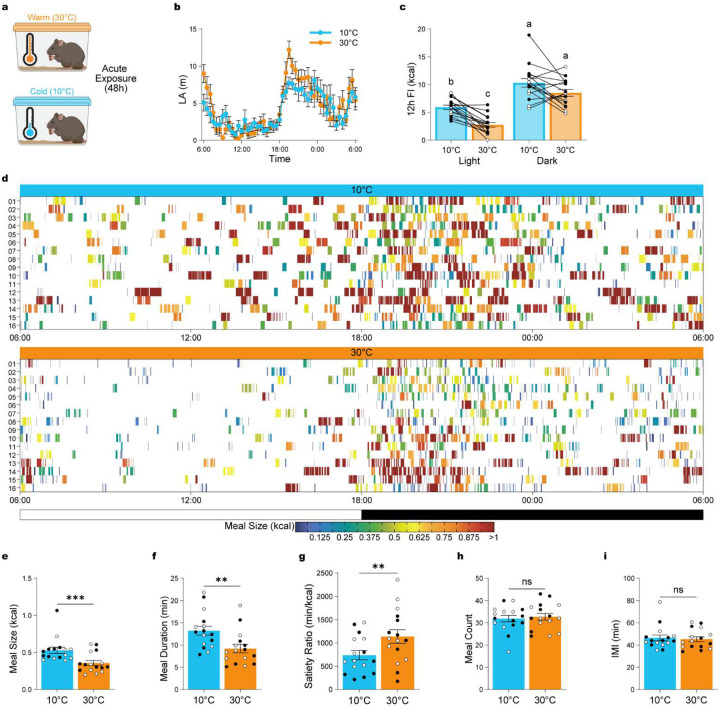
Temperature-dependent adaptations in meal patterns. (**a**) Schematic overview of acute 48h exposure to warm (30°C) versus cold (10°C) ambient temperatures. (**b**) Locomotor activity across 24 hours. (**c**) Total food intake analyzed by light/dark phases and temperature conditions (n = 16, 8 males, 8 females). (**d**) Heat map showing feeding events over 24 hours at 10°C and 30°C, with color-coded meal sizes. (**e**) Average meal size. (**f**) Average meal duration. (**g**) Satiety index (minutes of feeding per gram of food consumed). (**h**) Total number of meals. (**i**) Average inter-meal interval (IMI). Data are presented as mean ± SEM. Different letters above bars indicate statistically significant differences between groups. *p < 0.05, **p < 0.01, ***p < 0.001 (two-way ANOVA with Bonferroni’s post hoc test for B and I, t-test for C–H). Males are represented by black circles, females by white circles. IMI = inter-meal interval; LA = locomotor activity.

Most studies assess feeding by measuring total food consumed over time, however, feeding is a complex behavior composed of distinct components – such as meal size, frequency, duration, and satiety – and anatomically and functionally distinct neural circuits may regulate these behavioral components. To determine whether ambient temperature selectively alters specific aspects of feeding behavior, we performed detailed meal pattern analysis during 24h of cold (10°C; n = 16) or warm (30°C; n = 16) exposure (Figure 3a).

48h of warm or cold exposure did not result in significant alterations in LA (Figure 3b; repeated-measures ANOVA for the main effect of temperature: F_(1, 30)_ = 1.257, p = 0.2711). As expected, mice exhibited robust circadian feeding patterns at both temperatures (Figure 3c), with significantly more food consumed during the dark phase (light vs. dark phase: p_bonf_ < 0.0001), consistent with prior reports at room temperature^19,20^. Interestingly, the suppression of FI at 30°C was specific to the light phase (warm vs. cold during light phase: p_bonf_ = 0.0002), with no significant difference between temperatures during the dark phase (warm vs. cold during dark phase: p_bonf_ = 0.1024).

We further quantified and compared FI patterns across the 24h temperature exposure (Figure 3d). We found that warm exposure significantly reduced meal size (Figure 3e; paired t-test: t_(15)_ = 4.583, p = 0.0004) and meal duration (Figure 3f; paired t-test: t_(15)_ = 3.436, p = 0.0037). Additionally, there was an increase in satiety during warm temperature as indicated by an increased ratio of time between meals to amount of food eaten (Satiety ratio; Figure 3g; paired t-test: t_(15)_ = 3.182, p = 0.0062). In contrast, meal numbers (Figure 3h; paired t-test: t_(15)_ = 0.5005, p = 0.6240) and inter-meal intervals (IMI; Figure 3i; paired t-test: t_(15)_ = 0.4216, p = 0.6793) remain unchanged by temperature.

These findings demonstrate that warm ambient temperature selectively alters discrete features of feeding behavior – most notably meal size, duration, and satiety – while leaving meal frequency and timing intact.

### POA^Lepr^ neuron activation decreases meal size and increases satiety.

To determine whether activation of POA^Lepr^ neurons recapitulates the feeding behavior observed during warm exposure, we performed detailed meal pattern analysis following chemogenetic stimulation of this population. To avoid fluctuations in CNO levels from i.p. bolus injections and to minimize confounding effects from changes in LA, we first tested a range of CNO doses delivered via drinking water over 24h. A dose of 0.05 mg/kg/day was effective to suppress EE (Figure S2a) without altering LA (Figure S2b).

**Figure 4.**
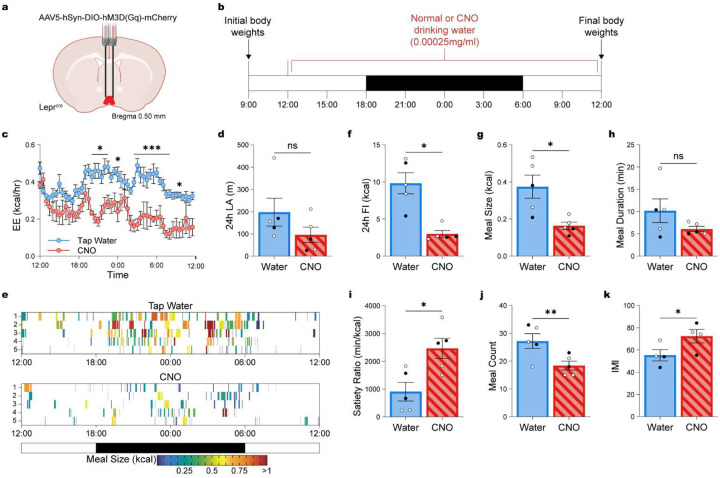
POA^Lepr^ activation recapitulates warm-temperature induced satiety. (**a**) Schematic depicting AAV5-hSyn-DIO-hM3D(Gq)-mCherry injection into the POA of Lepr^Cre^ mice. (**b**) Experimental design for continuous POA^Lepr^ activation. (**c–d**) A low-dose CNO regimen (0.00025 mg/ml) in drinking water; n = 5, 2 males, 3 females) significantly reduces energy expenditure (**c**) while maintaining locomotion for feeding by limiting body temperature drop (**d**) compared to mice given tap water. (**e**) Heat map of feeding events and meal sizes over 24 hours. (**f**) Total food intake over 24 hours. (**g**) Average meal size. (**h**) Average meal duration. (**i**) Satiety index. (**j**) Total meal count. (**k**) Average IMI. Data are presented as mean ± SEM. *p < 0.05, **p < 0.01, ***p < 0.001 (two-way ANOVA with Bonferroni’s post hoc test for C, t-test for D, F–K). Males are represented by black circles, females by white circles.

Mice with POA^Lepr^ specific DREADD-Gq expression (Figure 4a) received CNO in the drinking water (0.00025mg/ml) for 24h (Figure 4b). This treatment significantly reduced EE (Figure 4c; repeated-measures ANOVA for the effect of treatment: F_(47, 376)_ = 2.733, p < 0.0001), with no significant effect on LA (Figure 4d; paired t-test: t_(8)_ = 1.428, p = 0.1913). Consistent with preserved mobility, heat maps of individual feeding events confirmed that CNO-treated mice maintained consistent feeding patterns across the 24h period (Figure 4e). Chemogenetic activation of POA^Lepr^ neurons significantly reduced 24h FI (Figure 4f; paired t-test: t_(4)_ = 4.314, p = 0.0125).

Similar to warm exposure, POA^Lepr^ activation selectively reduced meal size (Figure 4g; paired t-test: t_(4)_ = 3.067, p = 0.0374) and increased satiety (Figure 4i; paired t-test: t_(4)_ = 2.838, p = 0.0470), while meal duration remained unchanged (Figure 4h; paired t-test: t_(4)_ = 1.874, p = 0.1343), though durations at 23°C (~10 min) were already shorter than those observed at colder temperatures (~12.5 min; see Fig. 3d). In contrast to warm exposure, POA^Lepr^ activation also decreased the total number of meals (Figure 4j; paired t-test: t_(4)_ = 5.522, p = 0.0053) and slightly increased IMI (Figure 4k; paired t-test: t_(3)_ = 3.365, p = 0.0436).

Together, these results demonstrate that activation of POA^Lepr^ neurons suppresses FI through selective modulation of feeding microstructure – most notably via reductions in meal size and increased satiety – supporting their role as warm-sensitive regulators of energy homeostasis.

### POA^Lepr^ neurons project to the melanocortin FI pathway and decrease FI in an energy-state dependent manner.

The POA is known to influence thermoregulatory behavior but its role in regulating FI – and the downstream circuitry mediating this effect – remains poorly defined. Prior work demonstrated that POA^Vglut2^ neurons contribute to temperature-dependent changes in FI^3^, with projection-specific activation revealing functionally divergent outcomes: some sites, such as the ARC, increase FI through engagement of AgRP neurons, while others, including the PVN and DMH, suppress FI in a manner consistent with warm-induced anorexia^3,4^. Because bulk POA^Vglut2^ activation recruits both warm- and cold-responsive populations, it remains unclear how these subtypes coordinate feeding behavior.

**Figure 5.**
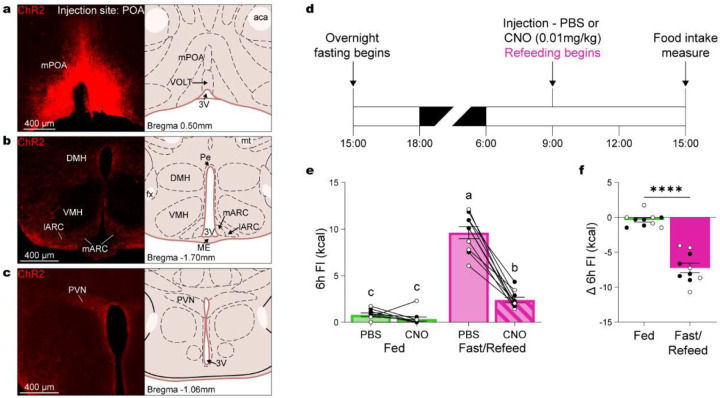
WS-POA^Lepr^ neurons project to satiety-related brain regions and suppress FI following refeeding. (**a**) Example of an injection site in the POA of a Lepr^Cre^ mouse injected with rAAV5/Efla-DIO-hChR2(H134R)-mCherry. (**b-c**) POA^Lepr^ neurons project to hypothalamic regions involved in satiety regulation, including the lateral arcuate nucleus (lARC) and dorsomedial hypothalamus (DMH) (**b**) and the paraventricular hypothalamus (PVN) (**c**). (**d**) Experimental timeline assessing the effects of chemogenetic activation of POA^Lepr^ neurons with CNO (0.01 mg/kg, i.p.) or PBS when mice are either fed (n = 10, 5 males, 5 females) or refed following an overnight fast (n = 10, 5 males, 5 females). (**e**) Total food intake over 6 hours. (**f**) Change in 6-hour food intake between PBS and CNO conditions. Data are presented as mean ± SEM. Different letters above bars indicate statistically significant differences between groups. *p < 0.05, **p < 0.01, ***p < 0.001 (two-way ANOVA with Bonferroni’s post hoc test for E, t-test for F). Males are represented by black circles, females by white circles.

To selectively trace the projections of warm-activated POA^Lepr^ neurons, we injected a cre-inducible EGFP reporter virus into the POA of Lepr^Cre^ mice (Figure 5a). Labeled axons were present in canonical feeding centers that included the DMH, ARC, and PVN (Figure 5b–c). Each of these regions are associated with distinct components of feeding regulation: the DMH modulates diurnal feeding rhythms^21^ and contains GABAergic Lepr neurons that inhibit AgRP cells to suppress FI^22^; the ARC harbors orexigenic POMC neurons involved in satiety, meal size, and duration^23–25^; and the PVN integrates POMC and AgRP input via MC4R-expressing neurons to regulate metabolic aspects of FI^26–28^.

Given these projection targets, we hypothesized that POA^Lepr^ neurons modulate FI through the melanocortin pathway. To test this, we examined whether POA^Lepr^ activation suppresses FI differently depending on energy state, specifically under conditions when melanocortin signaling is suppressed by fasting. Mice expressing DREADD-Gq in POA^Lepr^ neurons received a low dose of CNO (0.01 mg/kg) or PBS either after an overnight fast (just before refeeding) or while continuously fed. FI was measured over the subsequent 6 hours (Figure 5d). POA^Lepr^ activation significantly suppressed FI in fasted/refed mice but had minimal effect in continuously fed controls (Figure 5e; ANOVA food availability/treatment interaction: F_(1,_ 35) = 75.17, p < 0.0001). This energy-state-dependent effect was further supported by a comparison of net CNO-induced FI suppression, which was significantly greater in fasted/refed mice (Figure 5F; paired t-test: t_(9)_ = 9.621, p < 0.0001).

These findings indicate that the anorexigenic effects of POA^Lepr^ neurons are strongly dependent on energy state and most effective when melanocortin signaling is suppressed, supporting a functional interaction between POA^Lepr^ neurons and the melanocortin pathway in the regulation of FI.

### Pharmacologically induced satiety via melanocortin-4-receptors is temperature dependent.

Building on our findings that POA^Lepr^ neuron activation suppresses FI in a temperature-dependent manner and based on their projections to key nodes of the melanocortin pathway, we hypothesized that MC4R-expressing neurons may similarly mediate satiety at warm ambient temperatures. Prior work has shown that melanocortin signaling modulates meal size and satiety^23,25,27^, effects that closely resemble those induced by POA^Lepr^ activation. If MC4R neurons contribute to temperature-dependent regulation of FI, we reasoned that central activation of this pathway would suppress FI more effectively at cold versus warm ambient temperatures.

**Figure 6.**
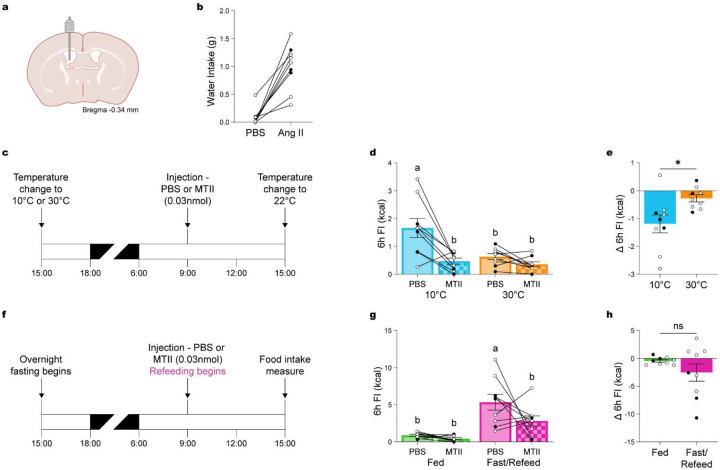
Temperature-dependent effects of pharmacologically induced satiety via melanocortin-4 receptor activation. (**a**) Schematic illustrating chronic intracerebroventricular (ICV) cannula implantation in mixed-background mice. (**b**) Two-hour water intake following Angiotensin II injection (50 ng, ICV) confirmed cannula placement. (**c**) Experimental timeline: MC4R agonist MTII (0.3 nmol, ICV) or PBS was administered during a 24-hour exposure to cold (10°C) or warm (30°C) temperatures (n = 9, 3 males, 6 females). (**d**) 6-hour food intake following MTII versus PBS injection. (**e**) Change in 6-hour food intake following MTII administration at different temperatures. (**f**) Experimental timeline assessing the effects of MTII (0.3 nmol, ICV) or PBS administration when mice are either fed or refed following an overnight fast (n = 9, 3 males, 6 females). (**g**) Total food intake over 6 hours. (**h**) Change in 6-hour food intake between PBS and MTII conditions. Data are presented as mean ± SEM. Different letters above bars indicate statistically significant differences between groups. *p < 0.05, **p < 0.01, ***p < 0.001 (two-way ANOVA with Bonferroni’s post hoc test for D, G, t-test for E, H). Males are represented by black circles, females by white circles.

To test this, we implanted ICV cannulas into the lateral ventricle of mixed-background mice (Figure 6a) and verified accurate placement using an angiotensin II-induced drinking test (Figure 6b)^29^. Mice were then housed overnight at either 10°C or 30°C and received an ICV injection of either the MC4R agonist Melanotan II (MTII; 0.3 nmol) or PBS the following morning (Figure 6c). MTII significantly suppressed 6h FI at 10°C, bringing intake down to levels comparable to those at 30°C (Figure 6d; ANOVA for temperature/treatment interaction: F_(1, 32)_ = 5.809, p = 0.0219). Moreover, the magnitude of MTII-induced FI suppression was significantly greater at 10°C compared to 30°C (Figure 6e; paired t-test: t_(8)_ = 2.990, p = 0.0173). These results support a role for MC4R signaling in mediating temperature-dependent suppression of FI and point to MC4R-expressing neurons as likely downstream effectors of POA^Lepr^-mediated satiety, although the precise site of action—whether in the PVN, DMH, or ARC—remains to be determined.

To further evaluate the context-dependence of MC4R-mediated FI suppression, we tested whether the effects of MTII differed based on energy state. Mice received an ICV injection of MTII (0.3 nmol) or PBS either after an overnight fast, just prior to refeeding, or while continuously fed. FI was measured over the following 6h (Figure 6f). Consistent with the energy-state-dependent effects observed following POA^Lepr^ activation, MTII significantly suppressed FI in fasted/refed mice but not in continuously fed mice (Figure 6g; ANOVA food/treatment interaction: F_(1, 32)_ = 2.603, p = 0.1165). Although the difference in the magnitude of FI suppression between groups did not reach statistical significance (Figure 6h; paired t-test: t_(8)_ = 1.177, p = 0.2730), the trend supports the notion that melanocortin-driven satiety is enhanced under conditions of elevated hunger. Together, these findings indicate that MC4R-mediated suppression of FI is both temperature- and energy-state-dependent, reinforcing the idea that POA^Lepr^ neurons engage melanocortin pathways to adaptively regulate feeding in response to environmental and physiological cues.

### POA^Lepr^ neuron activation increases POMC neuron activation in the anterior ARC.

The regulation of FI by POMC and AgRP neurons in the ARC is well established but their interaction with thermoregulatory circuits remains poorly defined. Recent work has shown that cold exposure robustly activates AgRP neurons^3,4^, whereas POMC neurons appear to be responsive to warm temperatures^17^.

**Figure 7.**
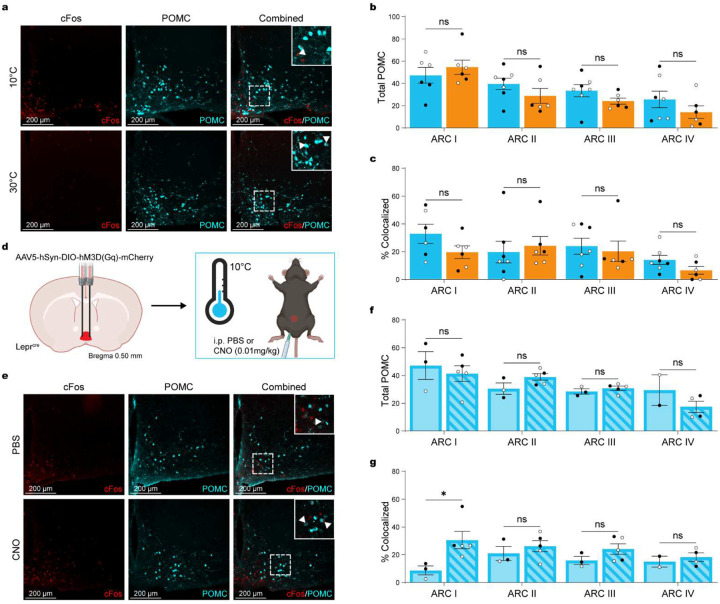
POA^Lepr^ activation increases POMC neuron activation in the anterior ARC. (**a**) Representative immunohistochemical images showing cFos (red), POMC (cyan), and cFos/POMC (merged) after 3-hour exposure to cold (10°C; n = 7, 3 males, 4 females) or warm (30°C; n = 6, 3 males, 3 females) in MC4R reporter mice. (**b**) Total POMC neuron count in the ARC. (**c**) Percentage of cFos+ neurons colocalized with POMC in the ARC. (**d**) Schematic depicting AAV5-hSyn-DIO-hM3D(Gq)-mCherry injection into the POA of Lepr^Cre^ mice. (**e**) Representative immunohistochemical images showing cFos (red), POMC (cyan), and cFos/POMC (merged) after CNO (0.01 mg/kg; n = 5, 2 males, 3 females) or PBS (n = 3, 2 males, 1 female) injection during 3-hour exposure to cold (10°C). (**f**) Total POMC neuron count in the ARC. (**G**) Percentage of cFos+ neurons colocalized with POMC in the ARC. Data are presented as mean ± SEM. *p < 0.05, **p < 0.01, ***p < 0.001 (two-way ANOVA with Bonferroni’s post hoc test). Males are represented by black circles, females by white circles.

We first tested whether warm ambient temperature alone activates POMC neurons by comparing cFos expression in POMC neurons following 3h exposure to 10°C or 30°C (Figure 7a). Neither the total number of POMC neurons (Figure 7b; ANOVA for the interaction of temperature and location: F_(3, 43)_ = 0.1.075, p = 0.3696) nor the percentage of cFos+/POMC+ colocalized cells (Figure 7c; ANOVA for the interaction of temperature and location: F_(3, 43)_ = 0.7723, p = 0.5159) differed between temperature conditions, suggesting that warm exposure alone is insufficient to drive POMC neuron activation.

Given the projection of POA^Lepr^ neurons to the ARC and our findings that MC4R activation suppresses FI, we next tested whether POA^Lepr^ neurons could drive POMC activation. To maximize the dynamic range for detecting activation, mice were cold-exposed for 3h (to reduce baseline POMC activity) and received i.p. injections of CNO (0.01 mg/kg) 1.5hrs before perfusions (Figure 7d–e). Total POMC neuron counts remained unchanged across groups (Figure 7f; ANOVA for the interaction of treatment and location: F_(3, 22)_ = 1.473, p = 0.2493). However, chemogenetic activation of POA^Lepr^ neurons significantly increased cFos expression in POMC neurons (Figure 7g; ANOVA for the main effect of treatment: F_(1, 18)_ = 6.545, p = 0.0198), with a significant increase in cFos/POMC colocalization in the rostral ARC (t-test with Bonferroni correction: t_(18)_ = 2.838, p_bonf_ = 0.0327).

These results support a model in which POA^Lepr^ neurons engage POMC neurons in the ARC to suppress FI, potentially through downstream activation of MC4R-expressing neurons.

### Warm ambient temperature decreased neuronal activation in PVN-MC4R neurons.

**Figure 8.**
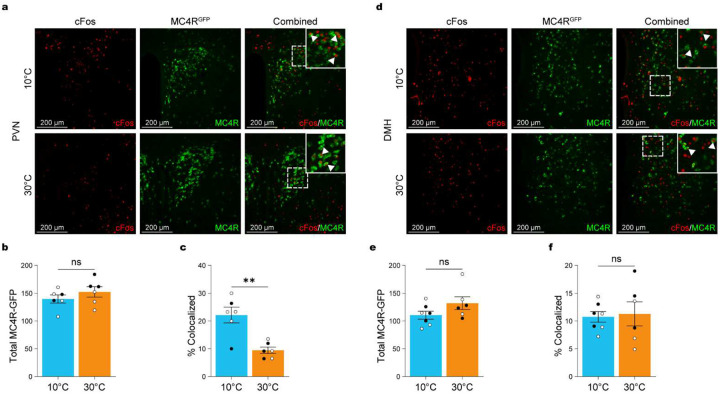
Warm temperatures reduce neuronal activation in PVN-MC4R neurons. (**a**) Representative immunohistochemical images showing cFos (red), MC4R-EGFP (green), and cFos/MC4R-EGFP (merged) in the PVN after 3-hour exposure to cold (10°C; n = 6, 2 males, 4 females) or warm (30°C; n = 6, 3 males, 3 females) in MC4R reporter mice. Insets show magnified regions. (**b**) Total MC4R neuron count in the PVN. (**c**) Percentage of cFos+ neurons colocalized with MC4R in the PVN. (**d**) Representative immunohistochemical images showing cFos (red), MC4R-EGFP (green), and cFos/MC4R-EGFP (merged) in the DMH after 3-hour exposure to cold (10°C; n = 7, 3 males, 4 females) or warm (30°C; n = 6, 3 males, 3 females) in MC4R reporter mice. Insets show magnified regions. (**e**) Total MC4R neuron count in the DMH. (**f**) Percentage of cFos+ neurons colocalized with MC4R in the DMH. Data are presented as mean ± SEM. *p < 0.05, **p < 0.01, ***p < 0.001 (t-test for B-E). Males are represented by black circles, females by white circles.

We next examined whether warm exposure modulates the activation of MC4R-expressing populations in downstream hypothalamic targets. MC4R^Cre^-GFP reporter mice were exposed to cold (10°C) or warm (30°C) ambient temperatures for 3h before perfusion (Figure 8a). We focused on the PVN and DMH, two hypothalamic regions that receive projections from POA^Lepr^ neurons (Figure 5), have a dense population of MC4R-expressing neurons and regulate feeding and energy balance^26,30,31^. Based on our prior findings that MC4R activation suppresses FI in a temperature- and energy-state-dependent manner, we hypothesized that warm exposure would preferentially engage satiety-promoting MC4R neurons in these downstream sites.

We first quantified the number of MC4R-expressing neurons in the PVN. No significant differences in total MC4R cell counts were observed between cold- and warm-exposed mice (Figure 8b; independent t test: t_(10)_ = 1.080, p < 0.3053). However, cFos analysis revealed significantly greater activation of PVN^MC4R^ neurons under cold conditions compared to warm (Figure 8c; independent t test: t_(10)_ = 4.183, p = 0.0019), indicating that ambient temperature dynamically regulates PVN^MC4R^ neuron recruitment.

We next examined MC4R-expressing neurons in the DMH (Figure 8d). Total MC4R neuron numbers in the DMH did not differ significantly between temperature conditions (Figure 8e; independent t test: t_(11)_ = 1.629, p = 0.1315). However, unlike the PVN, cFos activation of DMH^MC4R^ neurons was not significantly altered by temperature exposure (Figure 8f; independent t test: t_(11)_ = 0.2303, p = 0.8221). These findings suggest that DMH^MC4R^ neurons are not differentially recruited by acute changes in ambient temperature and highlight a selective role for PVN^MC4R^ neurons in mediating temperature-dependent neural and behavioral adaptations.

Together, these results reveal that among the MC4R-expressing populations examined, the PVN – but not the DMH – exhibits temperature-dependent activation.

### POA^Lepr^ neurons do not suppress medial ARC neurons but stimulate an unknown population of lateral ARC neurons.

**Figure 9.**
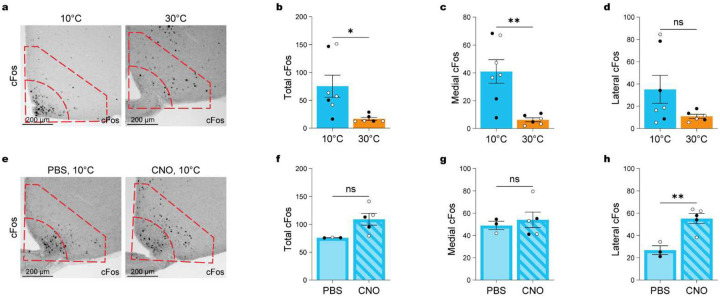
POA^Lepr^ activation does not inhibit cold-induced cFos expression in the medial ARC. (**a**) Representative immunohistochemical images showing cFos expression in MC4R reporter mice after 3 h of cold exposure (10°C; n = 6) and warm exposure (30°C; n = 10). Insets indicate the medial and lateral ARC regions used for quantification. (**b-d**) Quantification of cFos+ neurons in the total ARC (**b**), medial ARC (**c**), and lateral ARC (**d**) under cold and warm conditions. (**e**) Representative immunohistochemical images showing cFos expression after CNO (0.01 mg/kg; n = 5) or PBS (n = 3) injection during cold exposure. Insets show medial vs. lateral ARC regions. (**f-h**) Quantification of cFos+ neurons in the total ARC (**f**), medial ARC (**g**), and lateral ARC (**h**) following CNO vs. PBS injection during cold exposure. Data are presented as mean ± SEM. *p < 0.05, **p < 0.01, ***p < 0.001 (t-tests). Males are represented by black circles, females by white circles.

Given the increased activation of AgRP neurons during cold exposure^3,4^, we next investigated whether POA^Lepr^ activation affects ARC neuronal populations associated with hunger signaling. We first assessed cFos expression in the ARC following cold and warm exposure. Cold exposure significantly increased overall cFos-positive neurons in the ARC (Figure 9a–b; independent t test: t_(11)_ = 2.693, p = 0.0209), with anatomical mapping revealing that the increase was largely restricted to the medial ARC, where AgRP neurons are enriched^32^ (Figure 9c; independent t test: t_(11)_ = 3.750, p = 0.0032), while the lateral ARC showed no significant change (Figure 9d; independent t test: t_(11)_ = 1.777, p = 0.1033).

We next tested whether chemogenetic activation of POA^Lepr^ neurons suppressed cold-activated ARC neurons. Total cold-induced ARC cFos-positive counts were not significantly altered between PBS- and CNO-treated groups (Figure 9e–f; independent t test: t_(6)_ = 2.381, p = 0.0547). Surprisingly, the number of cFos-positive medial ARC neurons remained unchanged (Figure 9g; independent t test: t_(6)_ = 0.5144, p = 0.6254), indicating that POA^Lepr^ activation suppresses FI despite active AgRP neurons. Instead, we observed a significant increase in cFos-positive neurons in the lateral ARC (Figure 9h; independent t test: t_(6)_ = 4.225, p = 0.0055).

Although POMC neurons are typically located in the lateral ARC our prior mapping indicated that POA^Lepr^-induced POMC activation is biased toward the anterior ARC. Thus, these findings suggest that POA^Lepr^ neurons also engage an additional, unidentified lateral ARC population that may contribute to warm-induced FI suppression.

## 3. Discussion

Our work demonstrates that ambient temperature-induced adaptations of FI and EE are not fully balanced and significantly contribute to body weight changes. Chronic warm exposure is obesogenic, leading to increased adiposity in mice, whereas chronic cold exposure promotes fat loss. Chemogenetic activation of POA^Lepr^ neurons effectively mimics warm-suppressed feeding in a temperature- and energy-state-dependent manner, suggesting that leptin signaling influences feeding not only through classical homeostatic pathways (primarily ARC/PVN circuits) but also through thermoregulatory circuits involving the POA, DMH, and RPa.

MC4R neurons are central regulators of energy-state-dependent adaptations, and our data further shows that MC4R-mediated suppression of FI is also temperature- and energy state dependent. This supports a model in which POA^Lepr^ neurons modulate MC4R neurons to mediate warm-temperature induced FI suppression. Together, these findings highlight a critical role for POA^Lepr^ neurons in regulating body weight by actively modulating meal size and satiety through downstream melanocortin pathways. Notably, the clear interaction and integration of thermal and energy availability and their impact to change adiposity levels further suggests that homoeostatic setpoints may result from the integration of many environmental (exteroceptive) inputs.

Leptin is known to suppress homeostatic feeding but its efficiency is variable based on the body’s energy state with most effective FI suppression when leptin is applied during energy need states (fasting, food restriction) where endogenous leptin levels are low, and will enhance leptin induced suppression of FI during refeeding^33,34^. Our work extends these classical concepts by demonstrating that POA^Lepr^ induced FI suppression depends significantly on physiological states, shown here for thermal and energy states. Notably, leptin itself does not acutely alter EE or FI across temperatures^35^, and leptin deficient mice maintain an increased level of FI that does not change during warm or cold ambient temperature^36^. Similarly, selective deletion of Lepr in the POA has minimal effects on energy balance under baseline conditions. However, under homeostatic challenges, POA-specific Lepr deletion impairs energy balance^35^. In such cases, POA-specific Lepr knockout mice exhibited increased weight gain on a high fat diet and were protected from fasting-induced hypometabolism^35^. This suggests that POA^Lepr^ neurons are recruited under conditions that require adaptive homeostatic adjustments but otherwise have limited effects in unstressed states^36^.

Our findings align with a recent study showing that activation of POA^Vglut2^ neurons suppress FI^3^. This study, however, did not distinguish between cold- and warm-sensing populations of POA^Vglut2^ neurons. As a result, the temperature-dependent behavioral effects were a mix of warm-or cold-adaptive behaviors as demonstrated by projection-specific activation. Notably, POA^Lepr^ neurons are glutamatergic and warm-activated neurons^6^, characteristics further confirmed in single-nuclei RNA analysis in the POA^9^, supporting the use of POA^Lepr^ neurons for targeting a homogenic warm-sensing POA population. By focusing on POA^Lepr^ neurons, our study isolates a warm-sensing neuronal population and confirms their role in mediating temperature-dependent FI suppression. Whether POA^Lepr^ neurons are the only existing warm-sensing neuronal population is unclear, but other less restricted POA markers of warm-sensing neurons like transient receptor channel (TRPM2), PACAP/BDNF, prostaglandin receptor (EP3R) and opsin 5 seem to overlap with Lepr expressing neurons^37–40^ and explains similarly suppression of EE and body temperature. However, the variable potency of body temperature suppression compared to POA^Lepr^ activation suggests mixed warm- and cold-activated populations (PACAP/BDNF) or additional warm-sensing populations (TRPM2). Other POA populations like sleep inducing galanin neurons in the ventrolateral POA do not co-localized with Lepr^41^ but promote temperature-dependent changes in EE^42^, while activation of estrogen receptor alpha expressing neurons results in a slow onset of EE suppression^40^ supporting the idea that other populations might exist, even though their integration in homeostatic systems has not been studied in detail^43^.

We previously showed that POA^Lepr^ neurons are activated naturally at warm ambient temperatures^6,8–10^, and that chemogenetic activation at warm temperatures – when these neurons are already endogenously active – has little additional effect on EE^6^. Consistent with these findings, the current study shows that POA^Lepr^ activation robustly suppresses FI during cold but not warm conditions, reinforcing their temperature-dependent role. Moreover, both warm exposure and POA^Lepr^ activation suppressed FI by reducing meal size and duration while increasing satiety, indicating that warm temperatures primarily influence the meal termination phase of feeding^44,45^. Thus, our data demonstrates that these neurons play a key role in thermoregulatory feeding suppression, extending their key role in warm-induced feeding adaptations and the necessity to integrate into homeostatic feeding adaptations.

Prior studies have identified projections from the POA to the DMH, PVN, and ARC in temperature-dependent feeding regulation^3^, which we confirm in the present study. While POA^Vglut2^ projections to the DMH suppress FI, DMH circuits primarily regulate the appetitive stage (food seeking and meal initiation) via GABAergic projections to ARC^Agrp^ and ARC^POMC^ neurons^46,47^ and diurnal feeding rhythms^6,21,48,49^. Additionally, FI changes in response to warm and cold temperature were maintained consistently, not temperature-dependently, in the DMH^3^. In contrast, the PVN plays a central role in hunger regulation and satiety^50^. Prior research has shown that POA^Vglut2^ projections to the PVN decrease FI in a temperature-dependent manner^3^ and that the warm-induced decrease in FI is absent when central MC4R neurons are pharmacologically inhibited^18^, indicating that both direct and indirect POA > PVN activation may play a role in warm-induced FI suppression. This is consistent with our results where pharmacological activation of MC4R neurons with MTII suppressed FI predominantly at cold temperatures, indicating that temperature cues modulate the sensitivity of this pathway. However, we observed increased cFos expression in PVN^MC4R^ neurons with cold exposure compared to warm exposure, consistent with prior findings that PVN^MC4R^ activation increase EE and BAT thermogenesis^30,51,52^ but inconsistent with the findings by others that PVN^MC4R^ neurons are rather implicated in the control of FI not EE^26^. Thus, the role of PVN^MC4R^ neurons in temperature-dependent energy homeostasis needs further clarification in future studies.

POA^Lepr^ neurons are glutamatergic, so that the suppression of cold-activated POA^MC4R^ neurons is unlikely to be a direct activation of MC4R neurons. Thus, an indirect relay via the ARC is plausible. Prior studies show that cold-activated POA^Vglut2^ projections to the ARC increase AgRP neuronal activity and promote FI^3,4,7,16^ and cold-induced ARC^Agrp^ neuronal activation is required for cold-induced FI^16^. Importantly, we demonstrate that POA^Lepr^ activation suppresses FI potently during cold exposure, while cold-induced activation of AgRP neurons remains unchanged. Thus, FI suppression by POA^Lepr^ neurons overrides the powerful orexigenic drive of AgRP neuronal activation and must occur downstream of AgRP neurons. Instead, POA^Lepr^ activation induced cFos in anterior ARC^POMC^ neurons which could be a direct interaction of glutamatergic POA^Lepr^ neurons and suggests that the observed enhanced satiety is mediated through POMC-MC4R pathways. Indeed, POMC neurons reduce FI by projecting to PVN^MC4R^ neurons, and loss of POMC neurons increases meal size^23^, matching the feeding microstructure changes observed during warm exposure and chemogenetic POA^Lepr^ activation.

Although prior reports indicated warm temperature increases POMC activation^17,18,53^, we did not detect significant cFos/POMC colocalization after acute warm exposure. POMC neurons require prolonged activation to produce measurable decreases in FI^54–56^, and recruitment of POA neurons increases with extended heat exposure^57^. Also, POA^Lepr^ neurons are implicated in excessive heat adaptations^57^, thus even though we used extremely low chemogenetic activation doses we cannot rule out that this rather resembles a hot temperature rather than a thermoneutral temperature and would explain the lack of POMC activation at 30°C. Thus, acute warm exposure may not be sufficient to activate POMC neurons and explains why synthetic activation of POA^Lepr^ neurons is a more robust signal to activate POMC neurons.

Furthermore, we observed that POA^Lepr^ activation significantly increased cFos expression in non-POMC neurons within the lateral ARC. The identity of these neurons remains unknown, but they may represent additional populations involved in suppressing FI downstream of POA^Lepr^ activation. Future studies are needed to characterize these neurons and their contribution to thermoregulatory feeding adaptations.

Taken together, our findings highlight POA^Lepr^ neurons as critical integrators of environmental temperature cues that modulate feeding behavior and energy homeostasis. These neurons engage both thermoregulatory and classical homeostatic feeding circuits, particularly through interactions with the melanocortin system. POA^Lepr^ neurons thus represent an important node for adaptive energy balance regulation and may provide a promising therapeutic target for metabolic disease.

## 4. Materials and Methods

### Animal care

Male and female mice from the following strains were used in this study: C57BL/6J (JAX stock #000664), Lepr^Cre^ (B6.129-Lepr^tm3(cre)Mgmj^/J; JAX stock #032457)^58^, tg(POMC) (C57BL/6J-Tg(Pomc-EGFP)1Low/J; JAX stock #009593)^59^, and MC4R^Cre^ (Mc4r^tm3.1(cre)Lowl^/J; JAX stock #030759)^60^. Lepr^Cre^ mice and MC4R^Cre^ mice were crossed with EGFP-L10a (B6;129S4-Gt(ROSA)26Sor^tm9(EGFP/Rpl10a)Amc^/J; JAX stock #024750)^61^ or Ai95(RCL-GCaMP6f)-D (C57BL/6J) (B6J.Cg-Gt(ROSA)26Sor^tm95.1(CAG-GCaMP6f)Hze^/MwarJ; JAX stock #028865)^62^ to enable genetic labeling of specific neuronal populations. All experiments included both male and female mice unless otherwise noted. No sex differences were observed, and data from both sexes were pooled for analysis.

Mice were housed individually in standard ventilated cages with *ad libitum* access to laboratory rodent diet (#5001, LabDiet) and water unless otherwise noted. Animals were maintained on a 12-hour light/dark cycle (lights on at 0600 h) in a temperature- and humidity-controlled vivarium. All cages contained standard bedding and environmental enrichment, and husbandry practices adhered to institutional guidelines.

For experiments conducted under standard housing conditions, ambient room temperature was maintained at 23°C. For thermal challenge paradigms, mice were transferred to temperature-controlled environmental chambers (RIS77SD Rodent Incubator, Power Scientific Inc.; or DB034-LT-GD Laboratory Incubator, Darwin Chambers) set to 4°C, 10°C, 28°C, or 30°C for durations specified in each experimental protocol. Mice were acclimated to housing and handling for at least one week prior to experimentation. All animal procedures were approved by the Institutional Animal Care and Use Committee of Pennington Biomedical Research Center and complied with NIH guidelines for the care and use of laboratory animals.

### Stereotaxic viral injection

We performed stereotaxic viral injection of adenoassociated virus (AAV) as previously described^49^. We used 10–12-week-old Lepr^Cre^ mice for all surgeries. Mice were deeply anesthetized with 1–4% isoflurane and positioned in a stereotaxic alignment frame (#1900, David Kopf Instruments). Two viral constructs were used in this study: AAV5-hSyn-DIO-hM3D(Gq)-mCherry (a gift from Bryan Roth; Addgene viral prep #44361-AAV5; RRID: Addgene_44361) and rAAV5/Ef1a-DIO-hChR2(H134R)-mCherry (AV5214B; 4.0×10^12^ vg/mL), the latter obtained from the UNC Vector Core.

Bilateral injections were targeted to the POA using coordinates from the Paxinos and Franklin mouse brain atlas^63^: anteroposterior +0.55 mm, mediolateral ±0.25 mm, and dorsoventral −5.2 mm from Bregma. A total volume of 200–400 nL per side was delivered at a rate of 40 nL/min using a microinfusion system (UltraMicroPump III with MICRO2T, World Precision Instruments). After infusion, the injection needle was left in place for 5 minutes to minimize reflux. Craniotomies were sealed with bone wax (Lukens #901, Medline Industries), and the scalp was closed using wound clips (#203–1000, CellPoint Scientific).

Postoperative care included administration of warm sterile saline (1.5 mL, i.p.), carprofen (10–15 μL/g, s.c. daily for 72 hours), and lidocaine (100 μL, s.c. at the injection site) for analgesia. Mice were individually housed following surgery and allowed to recover for 3–4 weeks to ensure viral expression before experimental procedures.

Only animals with confirmed DREADD-Gq expression in the POA were included in the final analysis (n = 15). Mice with mistargeted or insufficient viral expression (n = 4) were excluded, as these animals failed to exhibit CNO-induced metabolic responses.

### Chemogenetic manipulations

For acute chemogenetic activation, mice received a single intraperitoneal injection of phosphate-buffered saline (PBS) or CNO (0.01 mg/kg, i.p.; freebase, HelloBio #HB1807) diluted in DMSO and PBS. Injections were administered following an 18-hour fasting period or 18 hours of temperature exposure, depending on the experimental paradigm. The selected dose was based on prior studies and pilot data demonstrating robust activation of POA^Lepr^ neurons with minimal off-target effects (see Supplementary Fig. S1A–C).

In a separate experiment, CNO was administered chronically via drinking water at a dose of 0.00025 mg/ml using water-soluble CNO dihydrochloride (HelloBio #HB6149). Mice were provided with CNO-containing water for a 24-hour period during which food intake and metabolic parameters were continuously monitored. Dosage was calculated based on an assumed average body weight of 25 g and estimated daily water consumption of 5 mL per mouse. This delivery approach allowed sustained activation of POA^Lepr^ neurons while minimizing potential systemic effects (see Supplementary Fig. S2A–B).

### Pharmacological manipulations via ICV injection

To manipulate central melanocortin receptor signaling, 10–12-week-old tg(POMC) mice were implanted with unilateral guide cannulas (C315G, Plastics One) targeting the right lateral ventricle. Stereotaxic coordinates relative to Bregma were: anteroposterior −0.3 mm, mediolateral −1.0 mm, dorsoventral −2.1 mm. Mice were anesthetized with 1–4% isoflurane, and cannulas were affixed to the skull with dental cement (C&B Metabond Quick, Parkell). Dummy cannulas (C315DC, Plastics One) were inserted postoperatively to prevent contamination or occlusion. Animals recovered for 3–4 weeks prior to experimental use.

To confirm accurate cannula placement, mice received a test ICV injection of angiotensin II (50 ng in 2 μL sterile saline; BACHEM #4474-91-3), and water intake was measured over 2 hours. A positive response was defined as the initiation of robust drinking behavior within 4 minutes post-injection. This test was repeated daily for up to three trials per animal. Mice that failed to respond were excluded from all subsequent experiments. Animals that passed the placement test were given a minimum of 3 days to recover before beginning the experimental protocol.

For pharmacological manipulation, mice were exposed to either a thermal challenge (10°C or 30°C for 24 hours) or fasted for 18 hours prior to refeeding. At the 18-hour time point, mice received a single ICV injection of either PBS or the MC4R agonist melanotan II (MTII; 0.3 nmol in 2 μL sterile PBS; BACHEM #4039778) via the implanted cannula. Injections were performed at 0900 h, and food intake was recorded after 6h (1500 h).

### Environmental temperature manipulations

For both acute and chronic thermal exposure experiments, mice were housed in temperature-controlled chambers (RIS77SD Rodent Incubator, Power Scientific Inc.) set to cold (4°C or 10°C), room (23°C), or warm (28°C or 30°C) ambient temperatures, depending on the experimental condition.

Chronic temperature exposures were conducted over a 4-week period, during which mice remained continuously housed at the assigned temperature. Body weight was recorded weekly, and body composition was assessed using nuclear magnetic resonance (NMR) spectroscopy (Minispec LF50/90 TD-NMR System, Bruker). In the final week, 24-hour food intake was measured to assess long-term effects of thermal environment on energy balance.

Acute thermal manipulations lasted either 3 or 24 hours. For 3-hour exposures, mice were placed in chambers at 0900 h and perfused at 1200 h for subsequent histological analyses. For 24-hour exposures, animals were transferred to the temperature-controlled chamber at 1500 h and remained there until 1500 h the following day. These temperature challenges were paired with pharmacological or chemogenetic interventions (CNO or MTII) or used as standalone stimuli prior to tissue collection, as specified in each experimental protocol.

### Feeding and fasting paradigms

For fasting-refeeding experiments, mice were food-deprived for 18 hours beginning at 1500 h. At 0900 h the following morning, animals received an intraperitoneal or intracerebroventricular injection of CNO or MTII, depending on the experimental protocol. Immediately following drug administration, food was returned to the cage to initiate the refeeding phase.

In fed control conditions, mice were maintained on ad libitum access to food prior to treatment. Food intake was measured manually at 6 hours post-refeeding (1500 h) to quantify acute effects on nutrient consumption.

### Metabolic phenotyping

Whole-body EE, LA, respiratory exchange ratio (RER), and FI were assessed using the Promethion metabolic phenotyping system (Sable Systems International). Mice were housed individually in sealed Promethion cages equipped with high-resolution temperature, activity, and gas-exchange sensors. Data were collected continuously over a 24- or 48-hour period, depending on the experimental protocol.

Temperature-controlled environmental chambers (DB034-LT-GD Laboratory Incubator, Darwin Chambers) housing the Promethion cages were set to either 10°C or 30°C to evaluate metabolic responses under cold or warm ambient conditions. In chemogenetic studies, mice were provided ad libitum access to water containing water-soluble CNO or control water during the 24-hour monitoring period. All metabolic chambers were calibrated before each trial, and data normalization was performed according to the manufacturer’s guidelines.

### Feeding behavior analysis

Meal patterns were derived from high-resolution food intake data using MacroInterpreter (Sable Systems International), which analyzes continuous weight measurements of the food hopper to identify discrete intake events. FI bouts were distinguished from non-consumptive fluctuations using the following criteria: a maximum inter-intake interval of 150 seconds, a maximum bout size of 1 g, and a significance threshold of *p* < 0.05. Total food consumed, as determined by bout analysis, was validated by correlating it with manually measured changes in hopper weight.

To define meals, intake bouts occurring within 5 minutes^19^ of each other were grouped using a custom R script. The satiety ratio was calculated as the interval between the end of one meal and the onset of the next (i.e., the intermeal interval), divided by the energy consumed during the preceding meal, as previously described^64^:

$$\frac{\text{time}\;\text{until}\;\text{next}\;\text{meal}}{\text{kcal}\;\text{consumed}\;\text{during}\;\text{a}\;\text{meal}}$$

The IMI was defined as the elapsed time between the start of two successive meals. FI was converted from grams to kilocalories by multiplying the weight of each meal by 2.89 kcal/g, based on the nutritional content of LabDiet #5001.

### Histology and immunohistochemistry

Perfusions and immunohistochemical processing were performed as previously described^7^. Mice were deeply anesthetized with isoflurane and transcardially perfused with PBS, followed by 4% paraformaldehyde. Brains were post-fixed, cryoprotected in sucrose, and coronally sectioned at 30 μm using a sliding microtome (SM2000R, Leica).

Free-floating sections were processed for immunohistochemistry to visualize cFos and other molecular markers. Nuclear cFos immunoreactivity was visualized using a diaminobenzidine (DAB) reaction (#34065, Thermo Fisher Scientific) following incubation with a peroxidase-conjugated anti-rabbit IgG reagent (ImmPRESS HRP, Vector Laboratories #30118). All other proteins were detected using fluorophore-conjugated secondary antibodies. The following primary antibodies were used: rabbit anti-cFos (1:1000; Synaptic Systems #226003), rabbit anti-POMC (1:1000; Phoenix Pharmaceuticals #H-029-30), and chicken anti-GFP (1:1000; Abcam #AB13970). Secondary antibodies included donkey anti-rabbit IgG-Alexa Fluor 594 (1:500; Invitrogen #A21207) and donkey anti-chicken IgG-Alexa Fluor 488 (1:500; Life Technologies #532354).

After staining, sections were mounted on glass slides, coverslipped with ProLong^™^ Gold Antifade Mountant (#P36930, Invitrogen), and imaged using a fluorescence microscope (BX51, Olympus). Images were acquired with a digital camera (DP30BW, Olympus) under appropriate filter sets for fluorophore visualization or bright-field illumination for DAB.

### Estimates of cell counts

Quantification of cFos-positive cells was performed in anatomically defined regions of interest based on the Paxinos and Franklin Mouse Brain Atlas^63^. The PVN was analyzed in 2–3 sections spanning −0.83 to −1.07 mm from Bregma; the DMH in 3–4 sections spanning −1.55 to −1.91 mm; and the ARC in 7–8 sections from −1.31 to −2.15 mm. The ARC was further subdivided into four rostrocaudal zones: ARC I (−1.31 to −1.43 mm), ARC II (−1.55 to −1.67 mm), ARC III (−1.79 to −1.91 mm), and ARC IV (−2.03 to −2.15 mm). In the intermediate ARC (−1.55 to −1.91 mm), both medial and lateral subdivisions were analyzed separately.

For each animal, cFos-positive nuclei were quantified bilaterally in matched sections using automated spot detection in NIS Elements AR 4.5 software (Nikon). Cytoplasmic markers, including POMC and EGFP, were manually counted due to their diffuse labeling patterns. All quantification was conducted by an experimenter blinded to treatment condition.

### Anatomical tracing

To visualize the axonal projections of POA^Lepr^ neurons, Lepr^Cre^ mice received bilateral injections of AAV5-EF1a-DIO-ChR2(H134R)-mCherry (AV4314C; 6 × 10^12^ vg/mL; UNC Vector Core) targeted to the POA. Following 12 weeks of viral expression, mice were transcardially perfused, and brains were coronally sectioned at 30 μm using a sliding microtome (SM2000R, Leica).

Native mCherry fluorescence was imaged using a wide-field fluorescence microscope (BX51, Olympus) to visualize ChR2-expressing axons. Projection fields were mapped across brain regions and aligned to neuroanatomical landmarks defined in Paxinos and Franklin Mouse Brain Atlas^63^.

### Statistical analysis

All statistical analyses were performed using GraphPad Prism version 10.3.1 (GraphPad Software). Differences between groups were assessed using two-way analysis of variance (ANOVA) or unpaired Student’s *t*-tests, with appropriate post hoc corrections applied as indicated. Data are presented as mean ± standard error of the mean (SEM), and statistical significance was defined as *p* < 0.05. Full statistical details, including test type, *n* values, and *p*-values, are provided in the corresponding results sections.

Schematic figures were created using BioRender (BioRender.com). All graphs were generated in GraphPad Prism version 10.3.1, except for heatmaps in Figures 3 and 4, which were created using ggplot2 (via the Tidyverse package) in RStudio version 2024.12.0+467 or later running R version 3.6.0 or later.

## Supplementary Files

This is a list of supplementary files associated with this preprint. Click to download.

- 20250630tempdepFISupplementaryFigures_final.docx

## Data Availability

Raw images obtained by immunostaining are available upon reasonable request to the Corresponding Author. Code for processing feeding events and generating the related figures is available from https://github.com/MunzbeH/2025_published-code/tree/main All collected and processed data used to generate figure graphs and the summary of statistical analysis are provided in the Source Data files.

## Highlights:

- Chronic warm ambient temperature induces obesity in mice.
- POA^Lepr^ neurons mediate temperature and energy state dependent food intake suppression
- Warm temperature and POA^Lepr^ neuronal activation suppress meal size and induce satiety
- Melanocortin-4-receptors are downstream mediators of warm-suppressed feeding
- PVN^MC4R^ and ARC^POMC^ neurons contribute to warm-suppressed feeding
- POA^Lepr^ neurons suppress cold-induced feeding despite maintained ARC^AgRP^-like activation

## Significance Statement:

Understanding how the brain integrates environmental cues to regulate food intake is crucial for developing interventions for obesity. Here, we explore the effects of warm temperature on feeding patterns in male and female mice. Our study reveals that leptin receptor-expressing neurons in the preoptic area (POA^Lepr^ neurons) play a critical role in the obesogenic effect of chronic warm temperature. Even though POA^Lepr^ neurons robustly suppress food intake, this is offset by simultaneous suppression of energy expenditure in response to warm temperatures and enables weight gain. By selectively activating these neurons in mice, we demonstrate that POA^Lepr^ neurons suppress feeding behavior through connections with the melanocortin pathway, a key regulator of energy balance. These findings challenge traditional views that leptin acts solely in energy homeostasis and provide new insights into the neural circuits involved in thermoregulatory feeding and energy balance. This work significantly advances our understanding of the brain’s role in integrating thermal and metabolic signals to maintain energy homeostasis.

## Code Availability

Custom code used for the processing and visualization of feeding events is available at https://github.com/MunzbeH/2025_published-code/tree/main.
